# Building Digital Literacy in Older Adults of Low Socioeconomic Status in Singapore (Project Wire Up): Nonrandomized Controlled Trial

**DOI:** 10.2196/40341

**Published:** 2022-12-02

**Authors:** Nerice Heng Wen Ngiam, Wan Qi Yee, Nigel Teo, Ka Shing Yow, Amrish Soundararajan, Jie Xin Lim, Haikel A Lim, Angeline Tey, Kai Wen Aaron Tang, Celine Yi Xin Tham, Jamaica Pei Ying Tan, Si Yinn Lu, Sungwon Yoon, Kennedy Yao Yi Ng, Lian Leng Low

**Affiliations:** 1 TriGen — Trigenerational Homecare Singapore Singapore; 2 Population Health and Integrated Care Office Singapore General Hospital Singapore Singapore; 3 Department of Internal Medicine Singapore General Hospital Singapore Singapore; 4 Department of Internal Medicine National University Health System Singapore Singapore; 5 Department of Family Medicine National University Health System Singapore Singapore; 6 Wee Kim Wee School of Communication and Information Nanyang Technological University Singapore Singapore; 7 Department of Psychiatry National Healthcare Group Singapore Singapore; 8 Medical Education Office Duke-NUS Medical School Singapore Singapore; 9 Department of Respiratory and Critical Care Medicine Tan Tock Seng Hospital Singapore Singapore; 10 Medical Social Services Ng Teng Fong General Hospital Singapore Singapore; 11 Research and Translational Innovation Office SingHealth Community Hospitals Singapore Singapore; 12 Health Services and Systems Research Duke-NUS Medical School Singapore Singapore; 13 Centre for Population Health Research and Implementation SingHealth Regional Health System Singapore Singapore; 14 Division of Medical Oncology National Cancer Centre Singapore Singapore Singapore; 15 SingHealth Duke-NUS Department of Family Medicine Singapore Singapore; 16 Outram Community Hospital SingHealth Community Hospitals Singapore Singapore

**Keywords:** digital literacy, health determinants, COVID-19 pandemic, social distancing, older adults, loneliness, social connectedness, quality of life, well-being, digital inclusivity, web-based, information and communication technology

## Abstract

**Background:**

In a rapidly digitalizing world, the inability of older adults to leverage digital technology has been associated with weaker social connections and poorer health outcomes. Despite the widespread digital adoption in Singapore, older adults, especially those of lower socioeconomic status (SES), still face difficulties in adopting information and communications technology and are typically digitally excluded.

**Objective:**

We aimed to examine the impact of the volunteer-led, one-on-one, and home-based digital literacy program on digital literacy and health-related outcomes such as self-reported loneliness, social connectedness, quality of life, and well-being for older adults of low SES.

**Methods:**

A nonrandomized controlled study was carried out in Singapore between July 2020 and November 2021 involving 138 digitally excluded community-dwelling older adults aged ≥55 years and of lower SES. Older adults awaiting participation in the program served as controls. Older adults under the intervention were equipped with a smartphone and cellular data, underwent fortnightly to monthly digital literacy training with volunteers to learn digital skills, and digitally connected to their existing social networks. Primary outcome was the improvement in self-reported digital literacy. Secondary outcomes included improvements in University of California, Los Angeles 3-item loneliness scale, Lubben Social Network Scale-6, EQ-5D-3L and EQ visual analogue scale scores, and Personal Wellbeing Score.

**Results:**

There were significant improvements in digital literacy scores in the intervention group as compared to controls (mean difference 2.28, 95% CI 1.37-3.20; *P*<.001). Through multiple linear regression analyses, this difference in digital literacy scores remained independently associated with group membership after adjusting for differences in baseline scores, age, gender, education, living arrangement, housing type, and baseline social connectivity and loneliness status. There was no statistically significant difference in University of California, Los Angeles 3-item loneliness scale, Lubben Social Network Scale-6, Personal Wellbeing Score, or EQ-5D Utility and visual analogue scale score.

**Conclusions:**

This study adds to the growing research on digital inclusion by showing that a volunteer-led, one-on-one, and home-based digital literacy program contributed to increase digital literacy in older adults of low SES. Future studies should look into developing more older adult–friendly digital spaces and technology design to encourage continued digital adoption in older adults and, eventually, impact health-related outcomes.

## Introduction

### Background

In today’s rapidly digitalizing world, more than one-third of the population remain digitally unconnected [1], of which older adults have the least presence on the internet despite the biopsychosocial benefits brought about by digital technology [2,3]. A review of information and communication technology (ICT) interventions on reducing social isolation in older adults has demonstrated a positive impact on social support, social connectedness, and social isolation [4]. Moreover, ICT use also has a positive impact on health and well-being by contributing to fewer depressive symptoms and higher self-rated health and subjective well-being in older adults [5,6], with these relationships mediated by reduced loneliness [7]. Conversely, the lack of digital literacy can affect older adults’ ability to access health resources and is associated with social isolation and poorer health outcomes [8-11]. At the same time, results from some studies imply that ICT use might not always be related to improved mood, quality of social relationships, and well-being [12,13].

Notwithstanding, digitally exclusion in older adults is often correlated with lower socioeconomic status (SES) [14]. This is further exacerbated by the COVID-19 pandemic, where physical distancing measures and pandemic control policies have contributed to increased social isolation and loneliness [15-17] and were associated with adverse outcomes such as depression, social anxiety, cognitive impairment, and early mortality [18,19]. Given the promising positive impact of ICT use and the negative impact of digital exclusion on loneliness, social connectedness, health, and well-being for older adults, it is of pertinent interest to better understand how digital literacy can be effectively improved among older adults in Singapore and investigate its impact on health-related outcomes, especially in the midst of the COVID-19 pandemic.

### Digital Adoption in Singapore

Despite the widespread digital adoption in Singapore, older adults still face difficulties in adopting ICT [20] due to psychosocial or socioeconomic reasons. As an effort to improve digital literacy among older adults in Singapore, the Infocomm Media Development Authority in Singapore launched the Seniors Go Digital Program [21]. The program was designed to address digital access by providing subsidized smartphones and mobile data subscriptions to older adults of low SES. However, the program had a lower-than-expected impact on their target group as the program was put together rapidly to meet the urgent needs during the pandemic, but there were still a substantial number of older adults not reached [22]. Insights from older adults’ learning have suggested personalized approaches in a home environment to best encourage disadvantaged older adults to participate in learning [23].

### Objective

Cognizant that a more deliberate approach was needed to reach out to older adults of lower SES who are digitally excluded, TriGen, a voluntary organization, and the Singapore General Hospital collaborated with Infocomm Media Development Authority and senior activity centers in Singapore on a volunteer-led, one-on-one, and home-based digital literacy program, Project Wire Up. The pilot study aimed to contribute to the international literature on digital literacy and learning in older adults by examining the impact of the home-based digital literacy building program on (1) digital literacy and (2) self-reported loneliness, social connectedness, as well as other health-related outcomes such as quality of life and subjective well-being for older adults. We hypothesized that the program would result in (1) improved digital literacy and (2) reduced perceived loneliness and improved social connectivity, quality of life, and well-being in older adults.

## Methods

### Intervention: Project Wire Up

Project Wire Up was a volunteer-led, one-on-one, goal-directed, and home-based digital literacy program. The program adopted a three-pronged approach: older adults were (1) equipped with smartphones and internet connection; (2) trained by volunteers for 6 sessions (1 to 2 hours per session) over 3 months that were held in the older adults’ homes; and (3) digitally connected to existing social networks. Working with national agencies, under the program, older adults of lower SES who were not digitally equipped could purchase a smartphone at a one-off price of US $15 and a 1-year mobile data plan at US $4 per month. Digital skills training was conducted during the home visits by trained volunteers, who guided older adults through a tiered curriculum of increasing difficulty that could be tailored to the needs of older adults. At the base level, older adults were taught the basic use of the phone, such as making calls and sending messages, before progressing to other social telecommunication platforms (eg, *WhatsApp*) or entertainment platforms (eg, *YouTube*). More digitally savvy older adults were taught advanced smartphone functions such as accessing government websites, making purchases, or paying bills on the web [24]. At the end of the program, older adults would be connected to existing formal and informal networks through platforms such as mobile communication apps. Supplementary materials have been provided in Multimedia Appendix 1.

### Participant and Recruitment

A nonrandomized, waitlist-controlled design was carried out between July 2020 and November 2021 to evaluate the effects of a volunteer-led, one-on-one, and home-based digital literacy program among older adults of lower SES residing in Singapore. The inclusion criteria for the study were as follows: residents in the southeast region of Singapore; aged >55 years; belonging to lower SES (as indicated by residency in public rental housing or recipient of Public Assistance Scheme [25], which usually requires a per capita monthly household income of US $477 or less); and agreeable to partake in the digital literacy program for at least 2 visits or more. These older adults were generally digitally excluded. Our study intentionally reached out to these digitally excluded individuals by working with local older adult care service providers and community partners. The recruitment of participants involved phone calls and door-to-door outreach.

**Figure 1:**
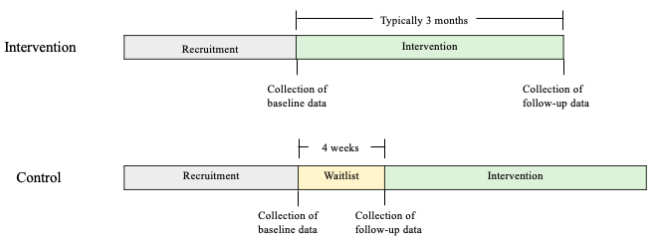
Overview of the participant’s journey with relation to data collection.

Upon agreement to join the program, participants were assigned to either the intervention or control arm using convenience sampling based on the referral timing to the program. For intervention group participants, baseline data were collected before exposure to the intervention, and follow-up data were collected after the completion of the intervention, which typically lasted for 3 months. As the study used a waitlist design, older adults in both the intervention group and control group were enrolled in the program, but for the control group, the baseline data were collected at the time of referral, and follow-up data were collected before exposure to the intervention, approximately 4 weeks after baseline data collection weeks (median 27, IQR 22-43 days; Figure 1).

**Figure 2:**
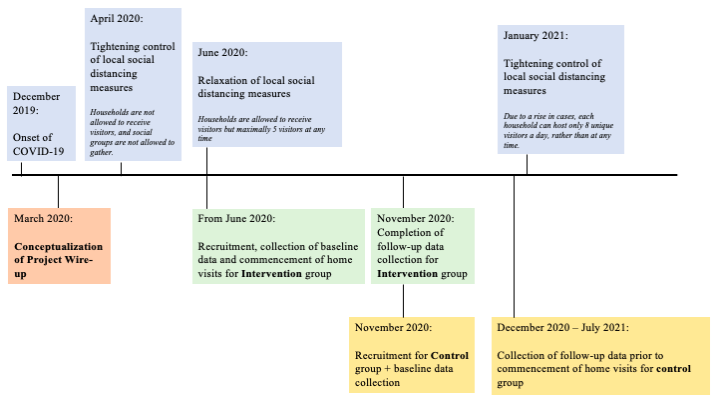
Timeline of the program with relation to policy changes in local social distancing measures.

Participants recruited from July 2020 to November 2020 were assigned to the intervention arm, whereas participants recruited from November 2020 to July 2021 were assigned to the control arm (Figure 2).

### Data Collection

Data were collected from participants either in person or via telephone through standardized self-reported questionnaires in participants’ preferred language. Standardized training was provided for surveyors prior to household visits for recruitment and survey administration. If there was no response at the first instance, visits or phone calls were conducted on at least 3 separate occasions, with at least 1 scheduled on a weekend, to maximize participation. Data collection was completed in November 2021.

### Measures

The primary outcome was digital literacy score, and secondary outcomes were Lubben Social Network Scale-6 (LSNS-6), University of California, Los Angeles 3-item loneliness scale (UCLA-3), EQ-5D, and Personal Wellbeing Score (PWS).

There is no universally accepted definition of digital literacy [26]. Hence, in this study, digital literacy is defined as the knowledge of the functional use of smartphones. To measure digital literacy, a 13-item self-reported digital literacy scale was constructed based on 4 domains of smartphone usage relevant and applicable to older adults [27]: Social (staying connected with social networks); Pass Time (using phones for relaxation or entertainment); Reassurance (feeling safe in an emergency); and Instrumental (obtaining news and information and accessing health, government, and banking services). An overall digital literacy score was computed by binarizing the scores (0=do not know how to use, 1=know how to use) and summed, with scores ranging from 0 to 13. The scale has been locally validated [28].

Social connectivity was measured using the locally validated LSNS-6 [29], where a higher numerical score indicates greater social connectedness [30]. Perceived loneliness was assessed using the UCLA-3, where participants scoring from 3-5 were classified as “not lonely,” whereas those scoring from 6-9 were classified as “lonely” [31]. Subjective well-being was assessed using PWS [32]. Quality of life was assessed using locally validated EQ-5D-3L and EQ visual analogue scale [33-35].

### Data and Statistical Analysis

Power analysis for sampling size was not calculated prior to the study as this was a pilot study. The aim of the study was to recruit at least 100 older adults in total.

Analysis was by intention to treat. Participant characteristics in both intervention and control groups were described by frequencies and their proportions for categorical variables and by means and 95% CI for numerical data. Independent sample 2-tailed *t* test, Wilcoxon sign-rank test, and *χ*^2^ tests were used to compare differences in baseline characteristics between participants of the different groups. Paired sample 2-tailed *t* test and Wilcoxon sign-ranked test were conducted to assess differences in participants characteristics and outcomes between baseline and follow-up for continuous variables within each group, dependent on the nature of data distribution within variables. Differences in loneliness statuses among participants between groups were explored by conducting a logistic regression analysis, adjusting for the baseline loneliness statuses of participants in the model.

Regression coefficients (*β*) and odds ratios of the association between group membership (control vs intervention) with the various outcome measures over time were estimated using a series of hierarchical linear or logistic regression models, dependent on the nature of the outcome variable in question. In these longitudinal analyses, the first model (Model 1) adjusted for baseline outcome scores/statuses. The second model (Model 2) adjusted for age, gender, education, housing type, and living arrangement at baseline, along with predictors in Model 1. In the third and final model (Model 3), social isolation and loneliness statuses at baseline were included as covariates alongside predictors indicated in Model 2. Statistical significance was set at *P*<.05 and tests were 2-tailed. Complete-case analysis was used for missing data. All analyses were conducted using STATA software (version 14; StataCorp LLC) [36].

### Ethics Approval

Ethical approval was obtained from SingHealth Centralized Institutional Review Board (2020/2722). Eligible participants provided written informed consent. The study follows the Transparent Reporting of Evaluations with Nonrandomized Designs (TREND) reporting guidelines [37].

### Results

#### Demographics

**Figure 3:**
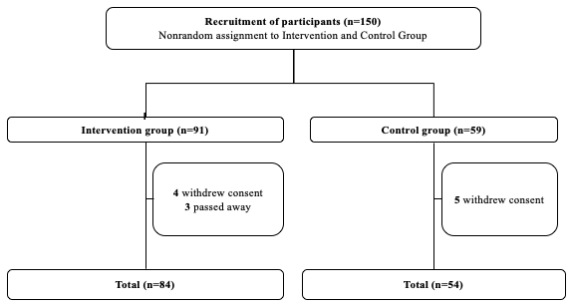
Consolidated Standards of Reporting Trials (CONSORT) flow diagram.

From July 2020 to November 2021, 150 older adults were invited to participate in the study. Of the 91 participants assigned to the intervention arm, 84 were included for analysis, with 7 participants excluded from analysis. Of the 59 participants assigned to the control arm, 5 were excluded from analysis, leaving 54 included for analysis (Figure 3).

Participants in both intervention and control groups were similar in age, gender, marital status, race, living arrangement, smartphone ownership, social connectivity, loneliness status, quality of life, and subjective well-being as seen in Table 1. Control group participants were found to have a significantly higher digital literacy score at baseline when compared to those in the intervention group (mean difference 2.28, 95% CI 1.37-3.20; *P*<.001). Participants in the intervention arm had a median of 3.5 (IQR 2-5) visits across the study duration.

**Table 1:** Participants’ baseline characteristics.

Characteristics		Intervention (n=84)	Control (n=54)	*P* value	
Age (years), mean (95% CI)		72.31 (70.38-74.24)	71.85 (69.86-73.85)	.74	
**Gender, n (%)**					.70
	Male	37 (44)	22 (41)		
	Female	47 (56)	32 (59)		
**Ethnicity, n (%)**					.57
	Chinese	67 (80)	40 (74)		
	Indian	8 (10)	5 (9)		
	Malay	9 (11)	9 (17)		
**Education, n (%)**					.53
	No formal education	23 (27)	12 (22)		
	Primary	34 (40)	23 (43)		
	Secondary	22 (26)	18 (34)		
	Diploma and higher	5 (6)	1 (2)		
Not married (single, separated, divorced, or widowed), n (%)		24 (29)	19 (35)	.41	
Living alone, n (%)		50 (60)	26 (48)	.19	
**Housing type A, n (%)**					.21
	Rental	66 (79)	47 (87)		
	Self-owned	18 (21)	7 (13)		
**Housing type B, n (%)**					.25
	1-room	52 (62)	30 (56)		
	2-room	21 (25)	20 (37)		
	3-room and above	11 (13)	4 (7)		
Have a mobile phone at baseline, n (%)		72 (86)	47 (87)	.83	
Have a smartphone at baseline, n (%)		51 (61)	39 (74)	.12	
*Digital literacy score, mean (95% CI)*		*3.77 (2.94-4.93)*	*5.09 (4.15-6.04)*	*.04*	
LSNS-6^a^ score, mean (95% CI)		11.07 (9.83-12.31)	12.36 (10.31-14.41)	.29	
Loneliness (UCLA-3^b^ score=6-9), n (%)		23 (27)	10 (19)	.23	
Personal Wellbeing Score (n=120), mean (95% CI)		8.42 (7.78-9.06)	7.47 (6.51-8.44)	.10	
EQ-5D Utility (n=135), mean (95% CI)		0.80 (0.75-0.85)	0.77 (0.69-0.85)	.74	
EQ-5D visual analogue scale (n=135), mean (95% CI)		66.79 (62.74-70.84)	70.54 (64.59-76.48)	.30	

^a^LSNS-6: Lubben Social Network Scale-6.

^b^UCLA-3: University of California, Los Angeles 3-item loneliness scale.

#### Primary Outcome

The intervention group observed a statistically significant difference in the change in their mean digital literacy score before and after program, as compared to those in the control group (mean difference in change: 2.28, 95% CI 1.37-3.20; *P*<.001; Table 2). Statistical control for this difference in baseline digital literacy scores was implemented in the analyses pertaining to the digital literacy score. Through multiple linear regression analyses, this change in digital literacy scores remained independently associated with group membership after adjusting for baseline digital literacy scores and differences in age, gender, education, living arrangement, housing type, and baseline social connectivity and loneliness status (Model 2, *β*=1.91, 95% CI 0.93-2.89; *P*<.001 and Model 3, *β*=1.90, 95% CI 0.91-2.90, *P*<.001), as seen in Table 3.

The domain-level analyses showed that the greatest gain was in the Instrumental domain (obtaining news and information and accessing health, government, and banking services), where the participants in the intervention arm learned, on average, approximately 1 more new function than the control arm, followed by the Reassurance, Social, and Pastime domains. The before and after program difference in all domains except for the Pastime domain remained statistically significant after controlling for covariates (Table 3).

**Table 2:** Intervention and control group differences.

Variable		Intervention, mean (95% CI)	Control, mean (95% CI)	Mean difference (95% CI)	*P* value
**Primary outcome analysis**					
	Digital literacy score	2.42 (1.73 to 3.11)	0.13 (–0.48 to 0.75)	2.28 (1.37 to 3.20)	<.001
**Domain-level analyses**					
	Social domain	0.64 (0.43 to 0.85)	0.20 (0.00 to 0.40)	0.44 (0.15 to 0.73)	.003
	Instrumental domain	0.77 (0.42 to 1.12)	0.04 (–0.28 to 0.36)	0.74 (0.27 to 1.20)	.002
	Reassurance domain	0.46 (0.24 to 0.07)	–0.15 (–0.40 to 0.07)	0.61 (0.30 to 0.92)	<.001
	Pastime domain	0.54 (0.29 to 0.78)	0.13 (–0.15 to 0.39)	0.41 (0.05 to 0.76)	.03

**Table 3:** Multiple linear regression analyses.

Variable		Model 1^a^		Model 2^b^		Model 3^c^	
		*β* (95% CI)	*P* value	*β* (95% CI)	*P* value	*β* (95% CI)	*P* value
**Primary outcome analysis**							
	Digital literacy score	1.99 (1.02 to 2.95)	<.001	1.91 (0.93 to 2.89)	<.001	1.90 (0.91 to 2.90)	<.001
**Domain-level analyses**							
	Social domain	0.34 (0.09 to 0.61)	.009	0.35 (0.09 to 0.61)	.009	0.35 (0.08 to 0.61)	.01
	Instrumental domain	0.66 (0.23 to 1.10)	.003	0.71 (0.26 to 1.15)	.002	0.70 (0.24 to 1.15)	.003
	Reassurance domain	0.32 (0.06 to 0.59)	.02	0.30 (0.03 to 0.57)	.03	0.32 (0.05 to 0.60)	.02
	Pastime domain	0.30 (–0.02 to 0.62)	.06	0.29 (–0.05 to 0.64)	.10	0.28 (–0.06 to 0.63)	.11

^a^Model 1: group (control [reference group] / intervention), baseline domain score.

^b^Model 2: predictors in Model 1 and age, gender, education, housing type and living arrangement at baseline.

^c^Model 3: predictors in Model 2 and social isolation (Lubben Social Network Scale-6) and loneliness (University of California, Los Angeles 3-item loneliness scale) at baseline.

#### Secondary outcomes

There was no statistically significant difference in LSNS-6 (mean difference –1.47, 95% CI –3.42 to 0.49; *P*=.14), EQ-5D Utility score (mean difference 0.09, 95% CI –0.02 to 0.2; *P*=.11) and visual analogue scale score (mean difference 1.20, 95% CI –6.11 to 8.52; *P*=.45), or PWS (mean difference –1.28, 95% CI –2.45 to –0.12; *P*=.69). Loneliness status, as measured by UCLA-3, showed no significant changes between the 2 groups before and after the intervention period (odds ratio 1.35, 95% CI 0.42-4.35, *P*=.62; Table 4).

**Table 4:** Secondary outcome analysis.

Variable	Intervention, mean (95% CI)	Control, mean (95% CI)	Mean difference (95% CI)	Odds ratio (95% CI)	*P* value
LSNS-6^a^	–1.26 (–2.58 to 0.06)	0.20 (–1.26 to 1.67)	–1.47 (–3.42 to 0.49)	N/A^b^	.14
EQ-5D Utility score	–0.07 (–0.15 to –0.002)	0.02 (–0.07 to 0.11)	0.09 (–0.02 to 0.20)	N/A	.11
EQ-5D VAS^c^ score	1.85 (–2.63 to 6.33)	3.06 (–2.81 to 8.92)	1.20 (–6.11 to 8.52)	N/A	.45
PWS^d^ total	–0.73 (–1.50 to 0.04)	0.56 (–0.34 to 1.45)	–1.28 (–2.45 to –0.12)	N/A	.69
Loneliness	N/A	N/A	N/A	1.35 (0.42 to 4.35)	.62

^a^LSNS-6: Lubben Social Network Scale-6.

^b^N/A: not applicable.

^c^VAS: visual analogue scale.

^d^PWS: Personal Wellbeing Score.

## Discussion

### Principal Findings

Our study revealed that a volunteer-led, one-on-one, and home-based digital literacy program undertaking a goal-directed approach contributed to a significant increase in digital literacy among community-dwelling older adults of low SES strata in Singapore who are digitally excluded. However, the program did not result in expected improvements in loneliness, social connectedness, quality of life, and personal well-being.

The increase in the knowledge of smartphone use in older adults of lower SES who are digitally excluded seen from our study could be attributed to key elements of our program [38]. The home-based, one-to-one approach to digital learning allowed us to better contextualize the digital training to each older adult while being able to provide close mentoring and support—factors found in adult learning literature to encourage ICT learning [23]. By breaking down the digital training into different tiers and matching each older adult’s capability, the program was able to build older adults’ confidence and sustain their motivation for smartphone learning, which has been shown to be important for technology adoption in older adults [39].

This increase in digital literacy did not translate into expected changes observed for loneliness, social connectedness, quality of life, and subjective well-being in our study. A possible explanation for this finding is that the older adults did not have any existing social networks to be tapped into and were at risk of social isolation as suggested by their LSNS-6 score being less than 12 [30]. Although deliberate efforts were made to digitally connect participants to their existing social networks, the participants’ limited social connections, a lack of corresponding digital adoption among peers in their social networks, and a dearth of social activities for older adults available on the digital space presented as challenges to the program. As such, the increase in digital literacy might not have translated to sustained use of new technology in older adults’ lives and or an increment in social activities or connections, resulting in a lack of observed changes for loneliness, social isolation, quality of life, and well-being. This finding is supported by studies in the literature that postulate that ICT use results in improvement in health-related outcomes in older adults by connecting them to their social networks, gaining social support, and engaging in activities of interest [4,40]

An implication from our study results is the need for digital literacy programs to move toward encouraging long-term digital adoption in older adults to truly impact health outcomes [22]. Future studies should look at the design of current web-based spaces and digital technologies, to develop more digitally inclusive spaces for older adults. This can increase the confidence and compatibility of digital technology with older adults, resulting in greater interest or motivations in older adults to take up digital technology [41] and the sustainability of digital literacy programs through continued use beyond programs [22].

### Strengths and Limitations

This study has several strengths. To the best of our knowledge, this is one of the first few studies in the world assessing the impact of a home-based digital literacy program on improving digital literacy among community-dwelling older adults of a low SES amid the COVID-19 pandemic. This provides vital empirical information required in the planning of future digital literacy programs for this vulnerable group in view of possible future pandemics.

Furthermore, data collection was conducted in person or via telephone interviews, unlike prior studies using web-based surveys to explore the effect of COVID-19 on older adults in other countries. This methodology allowed us to include participants who were digitally excluded and might not have been included in other web-based studies due to these older adults’ limitations or unwillingness to access the internet [42,43]. Through our study methodology, we were able to have a more representative picture of the impacts of our digital literacy program on older adults with little (or no) smartphone use during a pandemic.

At the same time, the design and implementation of our study was constrained by the practical limitations in implementing the intervention during the COVID-19 pandemic. The intervention group and control group participants were followed up at different time frames, as it would not be feasible to keep these control participants waiting beyond 4 weeks before being digitally equipped during the peak of the COVID-19 pandemic given the risk of potential social isolation. At the same time, using a waitlist design ensured that both groups were made up of individuals with the same inclination to participate in a digital literacy program, with the only difference between groups being the timing of the intervention. The delay for control group participants allowed the team to see the changes in digital knowledge and behavior across time when they are not participating in the digital literacy program. Follow-up data for the control group was specifically collected before they started the program to reduce confounding effects the training had on control participant’s digital literacy skills.

A nonrandom assignment of participants to groups was used due to the waitlist approach. A difference in baseline digital literacy score between groups was observed, where the control group had a higher baseline digital literacy score. To mediate this, statistical control for this difference was implemented in the analyses pertaining to the digital literacy score.

Finally, outcomes were self-reported by older adults, which may have impacted the accuracy in measuring changes in key outcomes such as digital literacy. Moving forward, future studies should use blinding of assessors and include objective assessments of the older adult’s digital literacy where practically feasible.

### Conclusion

Our study has provided preliminary empirical evidence to support the effectiveness of a volunteer-led, one-on-one, and home-based digital literacy program for older adults of lower SES in Singapore. Although the current intervention has a limited impact on secondary outcomes such as loneliness, social connectedness, quality of life, or subjective well-being, our findings is a step toward ensuring digital inclusivity in a world where there is rising inequity due to rapid digitalization. In the postpandemic world, digital use will no longer be a choice but an essential part of daily living [22]. For those who lack digital resources and know-how, their ability to access services and resources that impact the various determinants of health can be impeded, which can lead to adverse health outcomes [44]. Future studies should look into developing more age-friendly web-based spaces and technology design, which can encourage continued digital adoption in older adults and, eventually, impact health-related outcomes.

## Appendix

Multimedia Appendix 1 Compilation of all supplementary material inclusive of study protocol, questionnaires, training material, and Transparent Reporting of Evaluations with Nonrandomized Designs (TREND) checklist.

## Abbreviations

ICT: information and communications technology
LSNS-6: Lubben Social Network Scale-6
PWS: Personal Wellbeing Score
SES: socioeconomic status
TREND: Transparent Reporting of Evaluations with Nonrandomized Designs
UCLA-3: University of California, Los Angeles 3-item loneliness scale
