# Supplementary material for: Building Digital Literacy in Older Adults of Low Socioeconomic Status in Singapore (Project Wire Up): Nonrandomized Controlled Trial
Source: J Med Internet Res. 2022 Dec 2;24(12):e40341. doi: 10.2196/40341 (PMC9758632; doi:10.2196/40341)
Supplement: Multimedia Appendix 1 [file jmir_v24i12e40341_app1.docx]

**Appendix**

| ***Item*** | ***Page*** |
| --- | --- |
| Study Protocol | 3-8 |
| Pre-intervention Questionnaire | 10-14 |
| Post-intervention Questionnaire | 15-16 |
| Trend Checklist | 18-20 |
| Segments of the training material | 22 |

*This is a blank page.*

**STUDY PROTOCOL**

**INTRODUCTION**

**Background and Rationale**

Loneliness and social isolation are public health issues with economic, psychological and health repercussions. Social distancing is a public health policy used to control the spread of COVID-19 in the community but may exacerbate above issues^1^. While online platforms have mitigated the impact of social distancing for many, the uptake is poor amongst the older persons^2^.

A longitudinal survey in Singapore published in 2015 has shown that perceived loneliness increases the risk of all-cause mortality in the elderly^3^. With an ageing population in Singapore, it is estimated that more than half of Singapore residents aged 65 and above live alone or with their spouses only. A study by Duke-NUS noted that in 2009, about a quarter of people aged above 60 said they did not have a friend or a relative whom they could call on for help. This number doubled to 50 per cent in 2016^4^. To control the spread of COVID-19 in the community, multiple countries including Singapore have implemented social distancing measures^5,6^. While these measures are useful in containing the spread of the virus, there are economic, social and psychological repercussion.

**Objectives**

Project Wire-up is a voluntary program aiming to reduce social isolation and loneliness of elderly residents by increasing their connectivity with the community through online platforms. It aims to achieve this by equipping with hardware, improving information technology literacy and building a social network.

**Trial Design and study setting**

A non-randomized, waitlist controlled, design was carried out between July 2020 and November 2021 to evaluate the effects of a volunteer-led, one-on-one, home-based digital literacy program amongst older adults of lower SES residing in Singapore. The inclusion criteria for the study were as follows: residents residing in the southeast region of Singapore; above the age of 55; belonging to lower SES (as indicated by residency in public rental housing or recipient of Public Assistance Scheme^7^ which usually requires a per capita monthly household income of USD 477 or less) and were agreeable to partake in the digital literacy program for at least 2 visits or more. These older adults were generally a population of hard-to-reach individuals. In our pre-print study^8^, higher education levels and prior smartphone ownership independently increased the odds of a participant being willing to enroll in a DLP. Meanwhile lower digital literacy index and being Chinese (compared to non-Chinese) decreased the odds of being willing to enrol. Our study intentionally reached out to these traditionally hard to reach individuals by working with local eldercare service providers and grassroots to recruit older adults for the study. Recruitment of participants involved phone calls and door knocking in the community to reach out to these hard-to-reach individuals.

Upon agreement to join the program, participants were assigned into either intervention or control arm using convenience sampling based on the referral timing to the program.

For intervention group participants, baseline data was collected before exposure to the intervention and follow-up data after completion of the intervention, generally at the end of the three-month mark. (Figure 1). As the study employed a waitlist design, older adults in both intervention group and control group were enrolled in the program, but for control group, the baseline data was collected at the time of referral and follow up data before exposure to the intervention (Figure 1), approximately four weeks after baseline data collection weeks (Median: 27 days, 25^th^ percentile: 22 days, 75^th^ percentile: 43 days).

Participants recruited from July 2020 to November 2020 were assigned to the intervention arm, while participants recruited from November 2020 to November 2021 were assigned to the control arm.

**Eligibility Criteria**

The inclusion criteria for the study were as follows: residents residing in the southeast region of Singapore; above the age of 55; belonging to lower SES (as indicated by residency in public rental housing or recipient of Public Assistance Scheme^7^ and were agreeable to partake in the digital literacy program for at least 2 visits or more.

**Intervention**

 The program adopted a three-pronged approach: older adults were (1) equipped with smartphones and internet connection; (2) trained by volunteers for six sessions over three months; and (3) digitally connected to existing social networks. The digital skills training was facilitated by trained volunteers guiding older adults through a tiered curriculum of increasing difficulty, each tailored to the needs of older adults. Older adults were taught the basic usage of the phone such as making calls and sending messages. Many older adults progressed to use other social telecommunication platforms (e.g., *WhatsApp*), or entertainment platforms (e.g., *YouTube*). More digitally-savvy older adults were taught more advanced smartphone functions such as accessing government websites, and making purchases or paying bills online.

**Outcomes**

Primary outcome was digital literacy score, and secondary outcomes were LSNS-6, UCLA-3, EQ-5D, and PWB score.

To measure digital literacy, a 13-item self-reported digital literacy scale was constructed based on the four domains of smartphone usage that were relevant and applicable to older adults^9^; Social (to stay connected with social networks), Pass Time (to pass time, for relaxation or entertainment purposes), Reassurance (to feel safe in the event of an emergency) and Instrumental (to obtain news and information, accessing health, government and banking services).  An overall digital literacy score was computed by binarizing the scores (0=do not know how to use, 1=know how to use) and summed, with scores ranging from 0 to 13.

Social connectivity was measured using the locally validated Lubben Social Network Scale (LSNS-6)^10^. Perceived loneliness was assessed using the UCLA 3-item loneliness scale (UCLA-3), theoretical minimum and maximum score of 3 to 9, with a lower numerical score suggestive of loneliness^11^. Subjective wellbeing was assessed using Personal Wellbeing Score (PWS)^12^. Quality of life was assessed using EQ-5D-3L and EQ VAS^13-15^.

**Participant timeline**

Participants were trained by volunteers for six sessions over three months.


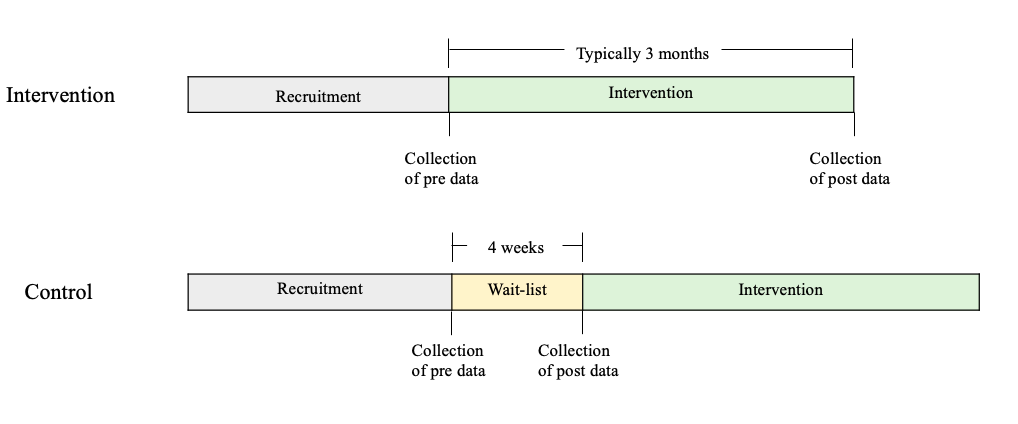


**Sample size**

No sample size was calculated prior to the study as this was a pilot study. The aim of the study was to recruit at least 100 seniors in total.

**Recruitment**

If the participant was agreeable to find out more about the project, the research assistant will begin reading the recruitment script. Consent for participation in the project was taken at this point if the patient was interested to participate. If the participant was not agreeable nor interested to participate, the participant was thanked for their time and no further attempts was made to recruit the participant.

**Allocation**

Participants were assigned into either intervention or control arm using convenience sampling depending on the timing of referral to the program; participants referred prior to November 2020 assigned to the intervention arm and those referred after November 2020 assigned to the control arm.

**Data collection methods**

For intervention group participants, home visits were initiated upon inclusion into the program, where baseline data was collected before exposure to the intervention and follow-up data after completion of the intervention. For control group participants, baseline data was collected at the time of referral and follow-up data was collected approximately four weeks after baseline.

Data was collected either by trained surveyors or the study team.

**Data management**

Data was downloaded and stored in an encrypted hard drive and stored within a locked area of the institution.

**Statistical methods**

Analysis was by intention to treat. Participant characteristics in both intervention and control groups were described by frequencies and their proportions for categorical variables, means and standard deviation (SD) for numerical data. Independent sample t-test, Wilcoxon sign-rank test and χ² tests were used to compare differences in baseline characteristics between participants of the different groups. Paired sample t-test and Wilcoxon sign-ranked test were conducted to assess differences in participants characteristics and outcomes between baseline and follow-up for continuous variables within each group, dependent on the nature of data distribution within variables. Differences in loneliness statuses among participants between groups were explored by conducting a Logistic Regression analysis, adjusting for the baseline loneliness statuses of participants in the model.

Regression coefficients (𝛃) and Odds Ratios (ORs) of the association between group membership (Control vs. Treatment) with the various outcome measures over time were estimated using a series of hierarchical linear or logistic regression models, dependent on the nature of the outcome variable in question. In these longitudinal analyses, the first model (Model 1) adjusted for baseline outcome scores/statuses. The second model (Model 2) adjusted for age, gender, education, housing type and living arrangement at baseline, along with predictors in Model 1. In the third and final model (Model 3), Social Isolation and Loneliness statuses at baseline were adjusted alongside predictors indicated in Model 2. Statistical significance was set at *p* < .05 and tests were 2-tailed.  Complete-case analysis was used for missing data. All analyses were conducted utilising the STATA Version 14^16^.

**Data monitoring**

The Principal Investigator performed data and safety monitoring.

**Harms**

This study had less than minimal risk. The Principal Investigator monitored and ensure no harm is afflicted upon to the elderly during the intervention.

**Auditing**

No auditing was carried out during the study but the data was monitored to ensure completeness of the data entries.

**Research ethics approval**

Ethical approval was obtained from SingHealth Centralized Institutional Review Board (2020/2722).

**Protocol amendments**

There was no protocol amendment made.

**Consent**

Consent was taken if the patient was agreeable to partake in the research project. If the participant was not agreeable nor interested to participate, the participant was thanked for their time and no further attempts was made to recruit the participant.

**Confidentiality**

Participants’ details were kept confidential during data analysis. The data will be maintained in the thumb drive or hard disk for a storage period of 7 years as suggested by CIRB.

**Declaration of interests**

Study was not sponsored by any organization.

**Access to Data**

Statistician and Research Administrators will have access to the data for data entry and analyses.

**Ancillary and post trial care**

Not applicable.

**Dissemination policy**

Not applicable.

**Citations**

1. Brooke J, Jackson D. Older people and COVID 19: Isolation, risk and ageism. Journal of Clinical Nursing. May 2020.
2. Armitage R, Nellums LB. COVID-19 and the consequences of isolating the elderly. The Lancet Public Health. 2020;5(5)
3. Ng TP, Jin A, Feng L, et al. Mortality of older persons living alone: Singapore Longitudinal Ageing Studies. BMC Geriatrics. 2015;15(1).
4. Visaria A, Malhotra R, Chan A. Changes in the Profile of Older Singaporeans: Snapshots from 2009 and 2016-2017. Singapore; 2019.
5. Lewnard JA, Lo NC. Scientific and ethical basis for social-distancing interventions against COVID-19. The Lancet Infectious Diseases. 2020.
6. Lee VJ, Chiew CJ, Khong WX. Interrupting transmission of COVID-19: lessons from containment efforts in Singapore. Journal of Travel Medicine. 2020;27(3).
7. Centre for Seniors. Comcare Long Term Assistance (Public Assistance Scheme) - Government Schemes Made Simpler. Accessed April 11, 2022. https://www.silverschemes.sg/post_single_age/comcare-long-term-assistance-public-assistance-scheme/
8. Soundararajan A, Lim JX, Ngiam NHW, et al. Smartphone Ownership, Digital Literacy, and the Mediating Role of Connectedness and Loneliness In Improving The Wellbeing among Community-Dwelling Singaporean Older Adults of Low Socio-Economic Status. Published online February 9, 2022. doi:[10.31234/osf.io/jx8b7](https://doi.org/10.31234/osf.io/jx8b7)
9. Wang Y, Matz-Costa C, Miller J, Carr DC, Kohlbacher F. Uses and Gratifications Sought From Mobile Phones and Loneliness Among Japanese Midlife and Older Adults: A Mediation Analysis. *Innovation in Aging*. 2018;2(3):igy027. doi:10.1093/geroni/igy027
10. Lubben J, Blozik E, Gillmann G, et al. Performance of an Abbreviated Version of the Lubben Social Network Scale Among Three European Community-Dwelling Older Adult Populations. *The Gerontologist*. 2006;46(4):503-513. doi:10.1093/geront/46.4.503
11. Steptoe A, Shankar A, Demakakos P, Wardle J. Social isolation, loneliness, and all-cause mortality in older men and women. Proceedings of the National Academy of Sciences. 2013;110(15):5797-5801. doi:10.1073/pnas.1219686110
12. Benson T, Sladen J, Liles A, Potts HWW. Personal Wellbeing Score (PWS)—a short version of ONS4: development and validation in social prescribing. *BMJ Open Qual*. 2019;8(2):e000394. doi:10.1136/bmjoq-2018-000394
13. Rabin R, Gudex C, Selai C, Herdman M. From Translation to Version Management: A History and Review of Methods for the Cultural Adaptation of the EuroQol Five-Dimensional Questionnaire. *Value in Health*. 2014;17(1):70-76. doi:10.1016/j.jval.2013.10.006
14. Merchant RA, Liu SG, Lim JY, Fu X, Chan YH. Factors associated with social isolation in community-dwelling older adults: a cross-sectional study. Qual Life Res. 2020;29(9):2375-2381. doi:10.1007/s11136-020-02493-7
15. Luo N, Wang P, Thumboo J, Lim YW, Vrijhoef HJM. Valuation of EQ-5D-3L Health States in Singapore: Modeling of Time Trade-Off Values for 80 Empirically Observed Health States. PharmacoEconomics. 2014;32(5):495-507. doi:10.1007/s40273-014-0142-1
16. StataCorp. *Stata Statistical Software: Release 14*. StataCorp LP.; 2015.

*This is a blank page.*

**PRE-INTERVENTION QUESTIONNAIRE**

Biodata

1. Abbreviation of participant’s name
2. Age
3. Gender
   1. Female
   2. Male
4. Race
   1. Chinese
   2. Malay
   3. Indian
   4. Eurasian
   5. Other
5. Marital status
   1. Single
   2. Married
   3. Separated
   4. Divorced
   5. Widowed
6. Current living arrangement
   1. Staying alone
   2. Staying with spouse only
   3. Staying with spouse and children
   4. Stay with children but not spouse
   5. Other
7. Religious belief
   1. Buddhist/Taosim
   2. Christian
   3. Islam
   4. Hinduism
   5. No religion
   6. Other
8. Employment status
   1. Fulltime
   2. Part-time
   3. Unemployed
   4. Retired
   5. Homemaker
   6. Other
9. Highest level of education
   1. No formal education
   2. Primary school
   3. Secondary school
   4. Diploma
   5. University Graduate
   6. Other
10. Housing type (a)
    1. Rental
    2. Self-owned
11. Housing type (b)
    1. One room
    2. Two room
    3. Three room
    4. Four room
    5. Other

**Usage**


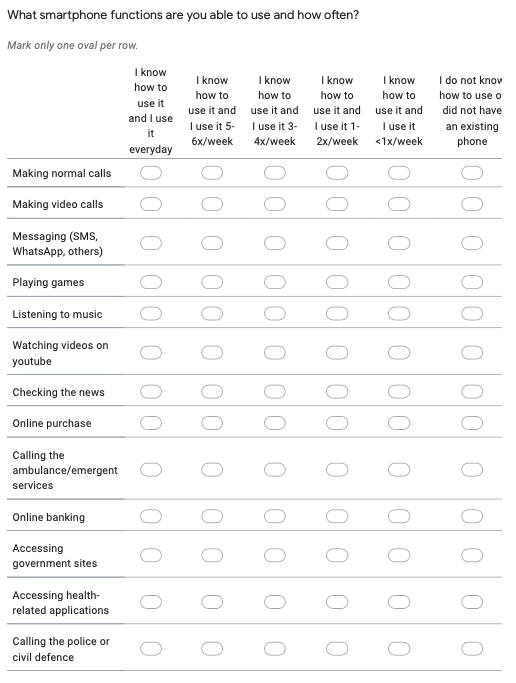


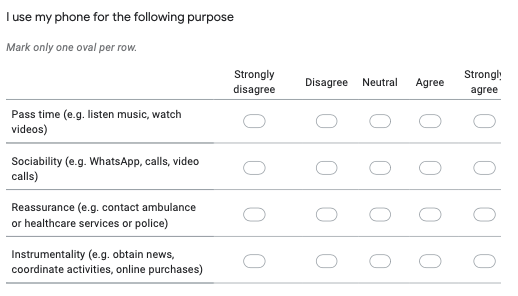


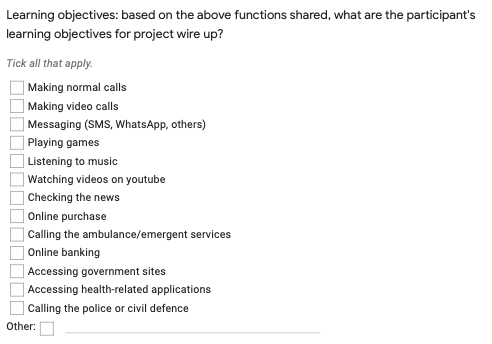


**Health and social outcomes**

1. UCLA-3 Point Loneliness Scale
2. Lubben Social Network Scale
3. EQ5D
4. Personal Well Being Score

**POST-INTERVENTION QUESTIONNAIRE**

**Usage**


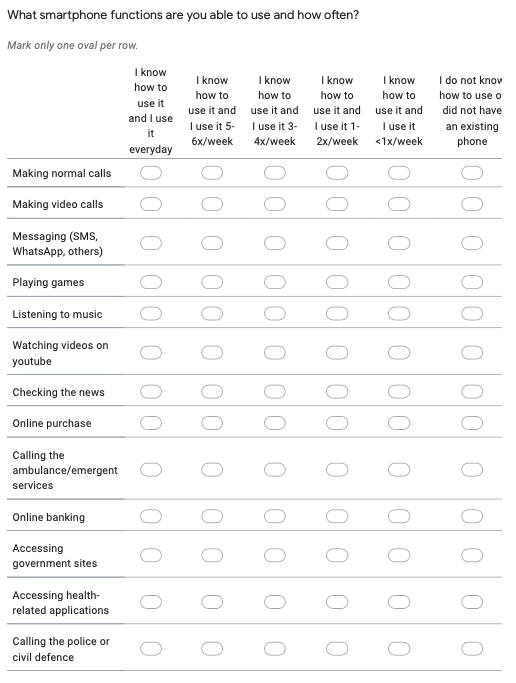


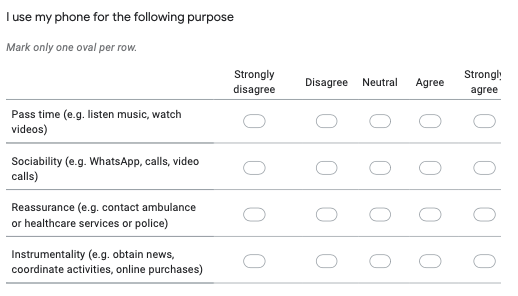


**Additional questions**

Minutes spent on the phone for the past 1 week

Data usage for the past 1 week

What difficulties did you face in using the phone?

How keen are you in continuing to use the phone after completion of wire up?

- Not very keen | Not keen | Neutral | Keen | Very Keen
- Why?

**Health and social outcomes**

1. UCLA-3 Point Loneliness Scale
2. Lubben Social Network Scale
3. EQ5D
4. Personal Well Being Score

**Feedback**

How can the wire up initiative be improved?
What do I wish he wire up initiative would have taught me?

*This is a blank page.*

**TREND CHECKLIST**

**
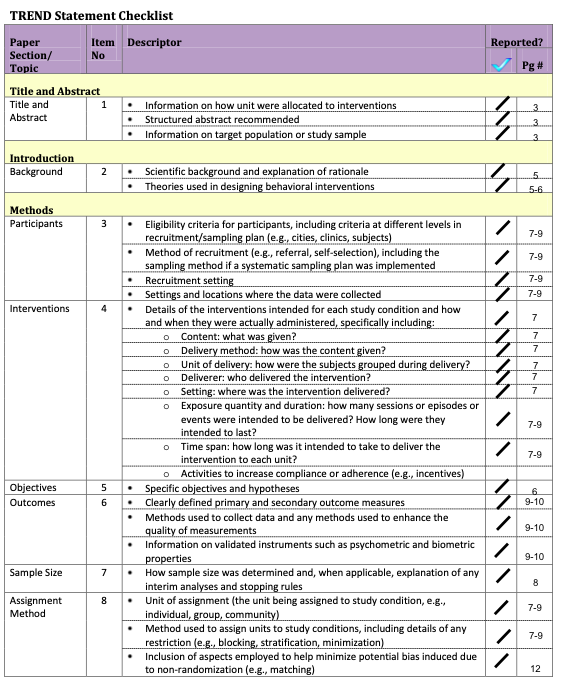
**

**
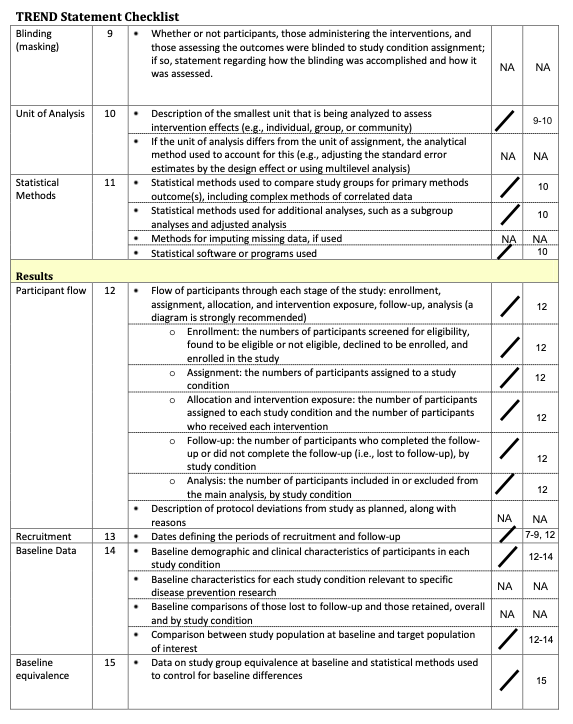
**

**
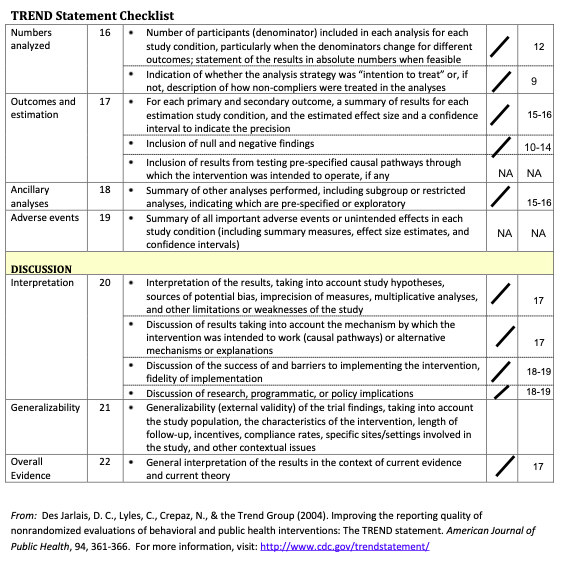
**

*This is a blank page.*

**TRAINING MATERIAL**

**Materials from Wire Up**

| Volunteer Training Manual | 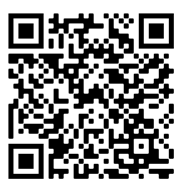 |
| --- | --- |

**Materials from Infocomm Media Development Authority (IMDA) Singapore**

| Starter Kit (online website) | https://www.imda.gov.sg/en/seniorsgodigital/Learn/Self-Learning/Starter-Kits |
| --- | --- |
| Starter Kit (pamphlet version) | https://drive.google.com/drive/u/0/folders/173k0D2FsVLx_8em5LOMK9WmHhOk1CsQf |
